# Long-Distance Runners and Sprinters Show Different Performance Monitoring – An Event-Related Potential Study

**DOI:** 10.3389/fpsyg.2018.00653

**Published:** 2018-05-08

**Authors:** Yuya Maruo, Timothy I. Murphy, Hiroaki Masaki

**Affiliations:** ^1^Department of Physical Education, Tokyo Women’s College of Physical Education, Kunitachi, Japan; ^2^Department of Psychology, Brock University, St. Catharines, ON, Canada; ^3^Faculty of Sport Sciences, Waseda University, Tokorozawa, Japan

**Keywords:** performance monitoring, error-related negativity, error positivity, Sport Competitive Anxiety Test, long-distance runner, sprinter

## Abstract

Previous findings have reported that track and field athletes may monitor and utilize internal information, including anxiety level, suggesting that the ability to inwardly monitor one’s own functioning and utilize anxiety are required to achieve superior performance. Performance monitoring has been investigated using two event-related potential components; the error (-related) negativity (Ne/ERN) and error positivity (Pe). It is unknown whether performance monitoring differs among various types of athletes. It has also been reported that Ne/ERN amplitude is increased in individuals who are more anxious and the prevalence and effect of anxiety also differs among various types of athletes. In this study, we recorded both Ne/ERN and Pe from long-distance runners (*n* = 24) and sprinters (*n* = 24) while they were performing a spatial Stroop task under motivation and no motivation conditions. We also collected scores on the Sport Competitive Anxiety Test (SCAT). Mean error rate on incongruent trials was lower in the motivation condition than in the no motivation condition. There was neither group effect, nor condition effect found in Ne/ERN amplitude. However, for the long-distance runners, Pe amplitude was larger in the motivation condition than in the no motivation condition. We also investigated the relationships between Ne/ERNs and individual differences in performance anxiety using the SCAT. A multiple linear regression analysis in the motivation condition revealed an interaction between type of runner and SCAT scores, indicating that long-distance runners with higher SCAT scores showed larger Ne/ERN amplitudes whereas the sprinters with high SCAT scores tended to exhibit smaller Ne/ERN amplitudes. Our findings provide further evidence that performance monitoring differs across various types of athletes.

## Introduction

It is well-known that the physical properties of muscles differ among various types of elite athletes. When comparing the muscles of sprinters and long-distance runners, distinct differences can be observed. In sprinters large numbers of fast-twitch muscle fibers are required to accelerate in a transient period, whereas for long-distance runners a greater number of slow-twitch muscle fibers are required to maintain their own pace during a relatively long-lasting race (Costill et al., 1976). Through long-term training, athletes learn to acquire not only specific physical functions related to performance characteristics; they also develop specific cognitive functions critical to maximize performance (Ripoll and Latiri, 1997; Nakamoto and Mori, 2012). Although previous studies have reported differences in muscle functions (Abe et al., 2000) as well as physiological functions (Itoh and Ohkuwa, 1990) between sprinters and long-distance runners, it remains unclear whether cognitive functions also differ between these two types of runners.

Previous findings support the notion that track and field athletes monitor and utilize internal information, including pace, fatigue and race image, during a competition (Morgan and Pollock, 1977; Mallett and Hanrahan, 1997). Morgan and Pollock (1977) found that elite marathoners were more aware of, and were better at monitoring their own level of fatigue compared to poorer marathoners. In addition, Mallett and Hanrahan (1997) found that elite sprinters carefully self-monitored their own performance, but were much less aware of their competitors. These findings suggest that the ability to inwardly monitor one’s own functioning is a necessary condition to achieve superior performance.

Performance monitoring, as currently conceptualized (Ullsperger and von Cramon, 2001) may be an essential cognitive function for improvement of motor skills (for a review see, Masaki and Sommer, 2012). Performance monitoring is associated with incorporation and examination of internal information and detecting errors between an actual ongoing movement and a desired movement. Performance monitoring has most often been investigated using two event-related potential (ERP) components. The first is the error negativity (Ne, Falkenstein et al., 1990) also referred to as the error-related negativity (ERN, Gehring et al., 1990) (referred to hereafter as the Ne/ERN). The second is the error positivity (Pe, Falkenstein et al., 1991). The Ne/ERN emerges over the frontocentral region approximately 70 ms after an erroneous response (Falkenstein et al., 1991), is thought to be generated by the anterior cingulate cortex (ACC) (Dehaene et al., 1994) and hence is maximal at FCz; whereas the Pe emerges over centroparietal regions approximately 200 to 500 ms after conscious error detection (Overbeek et al., 2005) and is maximal over Cz or Pz. Earlier studies have asserted that the Ne/ERN represents response conflict (Yeung et al., 2004) or error detection (Gehring et al., 1993). On the other hand, the Pe has been interpreted to reflect error evaluation and/or error awareness (Nieuwenhuis et al., 2001).

Recent studies have reported a relationship between aerobic capacity and performance monitoring processes. For example, Themanson and Hillman (2006) found that in individuals with high aerobic capacity the Ne/ERN amplitude was smaller but the Pe amplitude was larger than in those with low aerobic capacity. Because, it has been reported that aerobic capacity is higher in long-distance runners than in sprinters (e.g., Niemelä et al., 1980; Kusy and Zieliñski, 2015), it is presumed that the ERN amplitude should be smaller and the Pe amplitude should be larger in the long-distance runners compared to sprinters.

In this study, a spatial Stroop task was used to induce stimulus–response interference and thus response errors (Masaki and Segalowitz, 2004). We focused on error trials, and compared amplitudes of both the Ne/ERN and Pe between sprinters and long-distance runners who were members of a university track and field club.

It has also been noted that traits associated with emotion, such as anxiety, may influence performance, especially in long-distance runners. A field study examining long-distance runners reported that anxiety and motivation changed their pacing strategy in 1,600 m time trial races such that the lap time for the first 400 m was faster in the enhanced anxiety condition than in the reduced anxiety condition (Lane et al., 2016). Thus, it is likely that long-distance runners utilize anxiety and motivation to determine their pacing during this race. On the other hand, sprinters did not change their performance in a sprint task even in a highly anxious situation (Rathschlag and Memmert, 2015). According to these findings, we can presume that the relationship between anxiety and performance monitoring should differ between long-distance runners and sprinters.

Supporting a conceptualized notion that the ACC is involved in affective-emotional processes, it has also been reported that performance monitoring is influenced by anxiety and motivation (Bush et al., 2000). Hajcak et al. (2005) reported larger Ne/ERNs in a high incentive condition than in a low incentive condition, and concluded that the increases in Ne/ERN amplitude reflected the motivational significance of error. In addition, larger Ne/ERNs were also observed for individuals high in trait anxiety (Olvet and Hajcak, 2009a). These studies assumed that Ne/ERN amplitude is determined by the interaction between the motivational significance of the error and individual differences in trait anxiety (Proudfit et al., 2013). We hypothesized that the relationship between Ne/ERN amplitude and anxiety should differ between sprinters and long-distance runners, reflecting differences in how anxiety may affect their performance (Lane et al., 2016).

Previous studies asserted that the Pe reflects subjective error evaluation following error detection (Falkenstein et al., 2000). Endrass et al. (2010) reported a larger Pe in a monetary punishment condition compared to a control condition where there was no monetary penalty for errors. They suggested that Pe amplitude might reflect motivational evaluation of errors that was enhanced with monetary punishment. These findings appear to indicate that the Pe may represent subjective evaluation of errors. According to previous findings (Endrass et al., 2010), both the Ne/ERN and the Pe should be larger in high motivation conditions than in low motivation conditions.

Thus, we also wanted to investigate if the relationship between competitive anxiety and performance monitoring varies based on the type of athlete (i.e., long-distance runners vs. sprinters). We measured competitive anxiety from long-distance runners and sprinters, using the Sport Competitive Anxiety Test (SCAT; Martens, 1977). As can be seen above, it is highly possible that interactions among performance monitoring and individual differences in anxiety may be observed across different types of exercise (Thiel et al., 2012; Rathschlag and Memmert, 2015). If long-distance runners utilize anxiety to maintain their running pace during races (Thiel et al., 2012), the Ne/ERN amplitude for the long-distance runners should be larger for individuals higher in competitive anxiety than for those lower in competitive anxiety. On the other hand, sprinters performance is unrelated to anxiety (Rathschlag and Memmert, 2015); therefore, the Ne/ERN amplitude for the sprinters should not be associated with competitive anxiety. Thus, the relationship between the degree of competitive anxiety experienced in sports and performance monitoring might differ between the sprinters and long-distance runners. Specifically, we only expect a relationship between the Ne/ERN amplitude and anxiety for the long-distance runners.

## Materials and Methods

### Participants

Fifty-three participants (*Mage* ±*SEM* = 20.4 ± 0.2 years) were recruited from Waseda University’s Faculty of Sport Sciences. Five participants were excluded because they had fewer than six errors (Olvet and Hajcak, 2009b). We tested long-distance runners (*n* = 24) and sprinters (*n* = 24) who were members of a university track and field club. Participants had normal or corrected-to-normal vision and were paid 2,400 yen (about 28 U.S. dollars) for their participation. All participants gave written informed consent prior to the experiments. This study was approved by the Waseda University Ethics Committee.

### Questionnaire

Participants were administered the SCAT (Martens, 1977). The SCAT is a 15-item measure that assesses competitive trait anxiety. **Table 1** shows the SCAT scores in each group.

**Table 1:** SCAT scores (scores, SEM) in each group.

	SCAT
Sprinters	21.50 (0.77)
Long-distance runners	20.25 (0.79)

### Procedure

The participants rested both forearms and palms comfortably on a table to minimize any movements unrelated to their responses. We adopted a stimulus–response compatibility task, classified as a spatial Stroop task (Masaki and Segalowitz, 2004). A white fixation cross (0.7° × 0.7°) on a black background was continuously presented in the center of a computer monitor, placed 1 m in front of the participant. A white arrow (visual angle: 0.7° × 0.4°) pointing either up or down was shown above or below the fixation cross with an eccentricity of 0.8° visual angle (between center of fixation and arrow). Arrow direction (pointing up or down) and location (above or below fixation) were combined orthogonally, with each combination occurring equally often across participants. Trials where arrow direction agreed with arrow location (e.g., above fixation; pointing upward) were defined as congruent; trials where this was not the case (e.g., below fixation, pointing upward) were defined as incongruent.

Each trial began with a central fixation cross, shown for 300 ms; then, an arrow stimulus appeared either above or below the fixation for 150 ms. The arrow was followed by a blank screen for 1100 ms until the next fixation cross. Thus, the duration of each trial was 1400 ms. Participants were asked to respond both quickly and accurately with a brisk finger extension according to the pointing direction of the arrow (i.e., up or down), but not to its location. If participants did not respond within 600 ms, the feedback “Too Late!” was presented for 500 ms. Omitted responses were not regarded as errors, but excluded from analyses.

Responses were recorded with two microswitches mounted 150 mm apart in the mid-sagittal line. The microswitches were operated with small cantilevers that required an upward displacement for switch closure. A plastic plate (30 mm × 20 mm × 1 mm) was attached to the end of the cantilever key, providing leverage. Participants placed their middle fingers on the end of the plastic plate. The displacement of the key by lifting the finger resulted in switch closure and this was used as our definition of an overt response onset. We compared two conditions. In the motivation condition, each correct response was rewarded with a small amount of money (10 yen; about 12 cents), while participants lost 10 yen for each incorrect response. After the experiment, participants were told that their total could not become negative (i.e., below zero) in the motivation condition. Participants were given feedback about their current balance only at the end of each block – no feedback was given after individual trials. In the no motivation condition, participants would neither lose nor earn money and were not given any feedback regarding their performance.

In each condition participants performed four blocks of 72 trials each (288 trials in total). This resulted in a total of 72 trials for each combination of arrow direction and arrow location. Prior to the experiment participants practiced the task for 72 trials without any reward/punishment. The order of the two conditions and hand-to-key assignments were counter-balanced across participants.

### EEG Recording

The EEG was recorded from 128 sites with Ag/AgCl electrodes. Horizontal electrooculograms (hEOG) were recorded from the left and right outer canthi, and vertical electrooculograms (vEOG) from above and below the left eye. These were recorded with DC and 100 Hz low-passed filters, using the Biosemi Active Two system (Biosemi, Inc.). All physiological signals were digitized at 2048 Hz.

### Data Analysis

RT was measured as the interval between stimulus onset and microswitch closure. The error analysis reported here focused on incongruent trials (see section “Procedure”) because congruent trials resulted in were very few errors.

All ERPs were averaged, response-synchronized, using Brain Vision Analyzer. The EEG was re-calculated to an average common reference, and band-pass filtered 0.1–30 Hz (roll off 12 dB). Ocular artifacts were corrected using the procedure developed by Gratton et al. (1983). We excluded from averaging all trials in which response time was below 100 ms and where EEG voltages exceeded a threshold of 100 μV during the recording epoch. Percentage of exclusion from averaging was 0.3% in the motivation condition, and 0.4% in the no motivation condition, respectively.

A baseline of -400 to -300 ms prior to response was used. Ne/ERN amplitudes were scored at FCz as peak-to-peak amplitude by subtracting the most positive peak amplitude preceding the Ne/ERN from the negative peak amplitude of the Ne/ERN (Endrass et al., 2010). The negative peaks were determined within the time window of 100 ms following response onset. The positive peaks were determined within the time window of 100 ms preceding response onset. The Pe was measured at Cz as the mean amplitude in error trials between 200 and 350 ms after response onset.

Mean RTs and error rates were subjected to mixed three-way ANOVAs with repeated factors of Stimulus–Response congruency (congruent/incongruent), and Condition (motivation/no motivation) with Group (sprinters/long-distance runners) as a between group factor. Peak-to-peak Ne/ERN amplitudes (measured at FCz) and mean Pe amplitudes (measured at Cz) were subjected to a mixed two-way ANOVA with repeated factors of Condition with Group as a between factor. These sites for analysis were chosen based on previous research (e.g., Boksem et al., 2006) and examination of the topographic maps to determine where the effect was localized (see **Figure 2**). A Bonferroni correction was applied to *post hoc* comparisons. To investigate the effect of group on the relationship between SCAT scores and ERPs, we conducted a multiple linear regression analysis with SCAT scores, group, and the interaction SCAT scores × group separately for each condition. Cohen’s effect sizes were calculated to ensure the reliability of obtained results, adopting values of 0.02, 0.15, and 0.35 indicating small, medium, and large effect sizes, respectively (Cohen, 1992). To estimate how much a multiple linear regression analysis was sufficiently powered to detect significant difference, we conducted a power analysis using G^∗^Power 3 (Faul et al., 2007) and obtained power values 0.08, 0.33, and 0.67 for small, medium, and large effect sizes, respectively.

## Results

### Behavioral Data

#### Reaction Time

**FIGURE 1:**
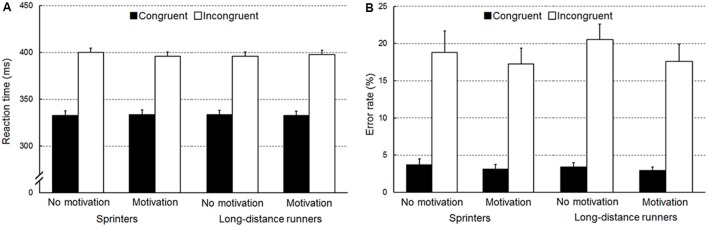
**(A)** Reaction time [ms, standard error of the mean (SEM)] and **(B)** the error rate (%, SEM) are presented separately for sprinters and long-distance runners.

**Figure 1A** shows the RTs. A three-way ANOVA for RTs revealed a three-way interaction among congruency, condition, and group [*F*(1,46) = 8.45, *p* = 0.006, $\eta_{p}^{2}$ = 0.16]. An additional two-way ANOVA restricted to incongruent trials revealed an interaction between condition and group [*F*(1,46) = 4.55, *p* = 0.04, $\eta_{p}^{2}$ = 0.09]. Simple effects analyses showed that sprinters tended to show shorter RT in the motivation condition than in the no motivation condition (*p* = 0.06). For congruent trials, no interaction between condition and group was found (*F* < 1).

A two-way ANOVA on RT with repeated measures of congruency and condition conducted only for sprinters showed an interaction between congruency and condition [*F*(1,23) = 8.30, *p* = 0.008, $\eta_{p}^{2}$ = 0.27]. Simple effects analyses showed that RTs on incongruent trials tended to be shorter in the motivation condition than in the no motivation condition (*p* = 0.09). The same two-way ANOVA conducted only for long-distance runners showed neither a main effect of condition (*F* < 1) nor an interaction (*F* < 1). RT of long-distance runners was significantly longer in the incongruent trials than in the congruent trials [*F*(1,23) = 470.72, *p* < 0.001, $\eta_{p}^{2}$ = 0.95]. In the no motivation condition, a mixed two-way ANOVA with a repeated measure of congruency revealed no interaction between congruency and group (*F* < 1). RT was significantly longer in the incongruent trials than in the congruent trials [*F*(1,46) = 862.20, *p* < 0.001, $\eta_{p}^{2}$ = 0.95]. In the motivation condition, a two-way ANOVA showed no interaction between congruency and group (*F* < 1). RT was significantly longer in the incongruent trials than in the congruent trials [*F*(1,46) = 887.84, *p* < 0.001, $\eta_{p}^{2}$ = 0.95].

#### Error Rate

**Figure 1B** shows error rates. A three-way ANOVA subjected to the error rate confirmed a significant interaction between condition and congruency [*F*(1,46) = 4.78, *p* = 0.03, $\eta_{p}^{2}$ = 0.17]. Simple effects analyses showed that error rate on incongruent trials was lower in the motivation condition than in the no motivation condition (*p* = 0.02). For congruent trials, there was no difference between the two conditions (*p* = 0.54). Neither a group effect nor an interaction was found (*F*s < 1).

### Response-Locked ERP

#### Ne/ERN

**FIGURE 2:**
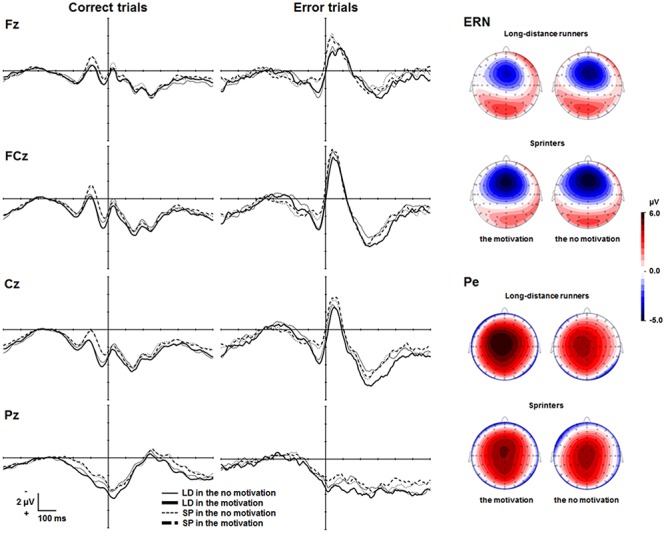
Response-locked grand averaged waveforms on error and correct trials at Fz, FCz, Cz, and Pz. Topographies (only for illustrative purpose) represent brain activities during the time windows ranging from 25 to 75 ms for the Ne/ERN and ranging from 200 to 350 ms for the Pe, respectively. LD, long-distance runners; SP, sprinters. Response-locked grand averaged waveforms on correct trials are shown only for illustrative purposes.

**Figure 2** depicts the response-locked ERP waveforms at Fz, FCz, Cz, and Pz. The frontocentrally distributed Ne/ERNs emerged about 50 ms after erroneous responses in both the motivation and the no motivation condition and were maximal at FCz. Mean amplitudes of the Ne/ERN for the long-distance runners in the motivation and the no motivation condition were -10.0 μV (*SEM* = 0.83), and -10.4 μV (*SEM* = 0.85), respectively. Mean amplitudes of the Ne/ERN for the sprinters in the motivation and the no motivation condition were -9.4 μV (*SEM* = 0.93), and -9.8 μV (*SEM* = 0.77), respectively. A two-way ANOVA with factors of condition and group showed no difference in ERN amplitudes between the two conditions (*F* < 1). Neither a group effect nor an interaction was found (*Fs* < 1).

#### Pe

**Figure 2** also shows Pe waveforms that emerged approximately 250 ms after erroneous responses and were maximal at Cz. Mean amplitudes of the Pe for the long-distance runners in the motivation and the no motivation condition were 5.8 μV (*SEM* = 0.68), and 4.2 μV (*SEM* = 0.63), respectively. Mean amplitudes of the Pe for the sprinters in the motivation and the no motivation condition were 4.6 μV (*SEM* = 0.74), and 4.6 μV (*SEM* = 0.80), respectively. A two-way ANOVA with factors of condition and group revealed a significant interaction between condition and group [*F*(1,46) = 4.06, *p* = 0.05, $\eta_{p}^{2}$ = 0.08]. Simple effects analyses revealed that the long-distance runners showed a larger Pe in the motivation condition than in the no motivation condition (*p* = 0.01), although the sprinters did not show any significant difference in Pe amplitudes between two conditions (*p* = 0.91). In addition, Pes did not differ between sprinters and long-distance runners in the no motivation condition (*p* = 0.70) or the motivation condition (*p* = 0.22).

### Multiple Linear Regression Analyses

**FIGURE 3:**
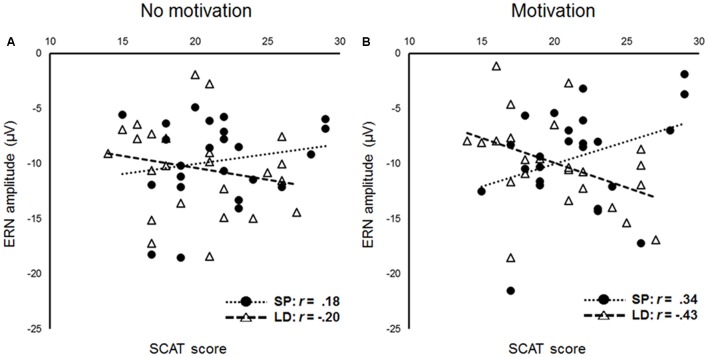
Scatter plots showing the relationships and correlations between scores on the SCAT and amplitudes of error-related negativity (ERN) [**A**, no motivation condition, **B**, motivation condition, circle: sprinters (SP), triangle: long-distance runners (LD)].

To investigate the effect of group on the relationship between SCAT scores and the Ne/ERN amplitudes, we conducted a multiple linear regression analysis with SCAT scores, group, and the interaction of SCAT scores × group as predictors separately for each condition. In the no motivation condition (**Figure 3A**), no interaction between SCAT scores and group was found (β = 0.27, *t* = 1.29, *p* = 0.21); however, in the motivation condition (**Figure 3B**), a significant interaction between SCAT scores and group was obtained (β = 0.53, *t* = 2.73, *p* = 0.01). Simple effects analyses showed that the long-distance runners with higher SCAT scores showed larger Ne/ERN amplitudes (β = -0.40, *t* = -2.05, *p* = 0.046). However, the sprinters with higher SCAT scores tended to exhibit smaller Ne/ERN amplitudes (β = 0.37, *t* = 1.82, *p* = 0.08).

The same analyses were conducted on Pe amplitudes but revealed no interaction between SCAT scores and the group in the either condition (motivation condition, β = -0.29, *t* = -1.39, *p* = 0.17; no motivation condition, β = -0.34, *t* = -1.67, *p* = 0.10).

## Discussion

We investigated if the differences in performance characteristics between sprinters and long-distance runners were associated with ERP indices of performance monitoring. Regardless of the type of runner, error rate was significantly lower in the motivation condition than in the no motivation condition. Because both speed and accuracy were emphasized and RT did not differ between conditions, this result is unlikely due to a spead-accuracy trade off. The lower error rate indicates that participants attempted to gain reward and avoid punishment in the motivation condition. This is consistent with previous reports that enhanced significance of an error decreased the error rate in a motivational situation (Hajcak et al., 2005; Maruo et al., 2016). Contrary to the behavioral results, the Ne/ERN did not differ between the motivation and the no motivation conditions, even though previous studies have reported larger Ne/ERNs associated with monetary reward and punishment (Hajcak et al., 2005). In addition, Ne/ERN amplitudes did not differ between sprinters and long-distance runners. Previous studies reported that smaller Ne/ERNs are associated with higher aerobic capacity (Themanson and Hillman, 2006; Pontifex et al., 2011). Our findings were not consistent with these previous studies. However, it is difficult to interpret our results according to these findings, because we did not directly measure aerobic capacity nor cerebellar gray matter volumes. Our results may also have been affected by a ceiling effect. Athletes in both types of running events have likely achieved a very rigorous error-detection system that was aqcuirred through multiple years of practice and competition and this may have contributed to a similar activation of the ACC in both groups.

To investigate the relationship between competitive anxiety and enhanced performance monitoring with monetary reward, we calculated correlations between SCAT scores and Ne/ERN amplitudes in the motivation condition. For the long-distance runners, individuals who had higher SCAT scores exhibited larger Ne/ERN amplitudes in the motivation condition in accordance with previous studies (Olvet and Hajcak, 2009a). However, sprinters with higher SCAT scores tended to exhibit smaller Ne/ERNs in the motivation condition. As we expected, the relationship between SCAT and Ne/ERN in the motivation condition differed between these two types of runners (i.e., sprint vs. long-distance). To our knowledge, this is the first demonstration of an effect of exercise type on the relationship between Ne/ERN and anxiety. Competitive anxiety may predict larger Ne/ERN amplitudes in the long-distance runners but not in the sprinters.

Many studies have confirmed that the Ne/ERN is associated with anxiety and negative affect (Olvet and Hajcak, 2009a; Aarts and Pourtois, 2010; Proudfit et al., 2013). For example, Aarts and Pourtois (2010) found larger Ne/ERNs for high-anxious than for low-anxious participants. Hajcak et al. (2004) also found a larger Ne/ERN in a high negative-affect group than in a low negative-affect group. According to these findings, a high anxiety trait is generally associated with a larger Ne/ERN. Our Ne/ERN results for the long-distance runnners are consistent with these previous findings. In this context, the results of the long-distance runners are expected and understandable.

Therefore, a weak relationship with higher anxiety being associated with smaller Ne/ERN for the sprinters might be a special case. Rathschlag and Memmert (2015) investigated whether or not self-generated emotion could enhance the performance of sprinters. Although induced anxiety did not influence running time in a 40 m sprint task compared to the emotionally neutral condition, induced happiness improved the running time. They also found that in the anxiety condition sprint time did not correlate with either trait or state anxiety as measured by the State-Trait Anxiety Inventory (STAI; Spielberger et al., 1970). Thus, the sprinters appeared to achieve performance skills relatively independent of trait anxiety. It is possible that the error detection system of sprinters is not noticeably influenced by competitive anxiety and they are not as concerned about a risk of error commission.

Long-distance runners, but not sprinters, showed a larger Pe amplitude in the motivation condition than in the no motivation condition. This result is consistent with previous findings. Endrass et al. (2010) found larger Pe amplitudes when they manipulated extrinsic motivation with monetary punishment, reflecting enhancement of subjective error evaluation. Maruo et al. (2016) also showed that the Pe increased in amplitude with either monetary reward or punishment. Thus, it is reasonable to conclude that long-distance runners experienced enhanced conscious error-evaluation with increased extrinsic motivation. By contrast, the Pe amplitude in sprinters was not increased in the motivation condition. The interaction between exercise types and the motivation condition suggests that long-distance runners and sprinters may show different motivational evaluations of errors (Endrass et al., 2010). Although Themanson and Hillman (2006) reported that the Pe amplitude was larger in individuals with high aerobic capacity than in those with low aerobic capacity, the Pe amplitude did not significantly differ between sprinters and long-distance runners.

It should be noted that our study has some limitations. First, we did not test non-athletes as a control group. Comparing track-and-field athletes with a group of non-athletes may have helped further clarify differences among long-distance runners, sprinters, and non-athletes. Second, if we had directly measured aerobic capacity from participants we could have reconfirmed the differences in aerobic capacity between long-distance runners and sprinters previously reported by others (Niemelä et al., 1980; Kusy and Zieliñski, 2015). Third, we did not find any significant correlations between SCAT scores and Pe amplitudes. In accordance with a previous finding (Proudfit et al., 2013), this result suggests that the error evaluation process might not be influenced by anxiety.

In sum, we found that Pes in the long-distance runners are modulated by the affective-motivational significance of errors, suggesting that long-distance runners may thoroughly evaluate their own errors in a motivational situation. We also found that in the motivation condition the long-distance runners with higher competitive anxiety showed larger Ne/ERNs, whereas the sprinters with higher competitive anxiety tended to exhibit smaller Ne/ERNs. These results suggest a particularity associated with long-distance runners in terms of their reaction to errors. Taken together, our findings may provide further evidence that the relationship between performance monitoring and individual differences in anxiety may differ across various types of sports.

In order to maximize the effectiveness of athlete’s training and subsequent performance in competition it is important for coaches and athletes to understand all aspects of their performance, both physically, and cognitively. Our results suggest that different types of athletes may differ cognitively and utilize performance monitoring in different ways. If sprinters and long-distance runner not only have significant differences in muscle structure but also brain structure/performance this may be an important consideration in terms of how athletes train. Further research in this area will be required to enhance our understanding of the cognitive differences among various types of athletes in order to optimize each individual performance.

## Ethics Statement

This study was carried out in accordance with the recommendations of human research of guidelines, the Ethics Review Committee on Research with Human Subjects of Waseda University with written informed consent from all subjects. All subjects gave written informed consent in accordance with the Declaration of Helsinki. The protocol was approved by the Ethics Review Committee on Research with Human Subjects of Waseda University.

## Author Contributions

YM and HM: designed the experiment and analyzed data. YM: performed the experiment. YM, TM, and HM: interpreted the data and wrote the paper. All authors made direct contribution to the work and approved it for publication.

## Conflict of Interest Statement

The authors declare that the research was conducted in the absence of any commercial or financial relationships that could be construed as a potential conflict of interest.
